# Disseminated sporotrichosis with orbital osteomyelitis in a child: a case report

**DOI:** 10.1590/S1678-9946202668046

**Published:** 2026-07-24

**Authors:** Fabiana Bononi do Carmo, Kimberly Souza Rocha, Caio Vinícius Luís, Aída de Fátima Thomé Barbosa Gouvea, Daisy Maria Machado, Ana Isabel Melo Pereira Monteiro, Érica Regina Cruz Paulino, Marina Gomes Pereira Sardinha, Kelly Simone Almeida Cunegundes, Paulo Gois Manso, Anderson Messias Rodrigues, Pedro Fiorini Pucchini, Marcelo Luiz Abramczyk, Maria Aparecida Gadiani Ferrarini, Carolina Sanchez Aranda Lago, Regina Célia de Menezes Succi

**Affiliations:** 1 Universidade Federal de São Paulo, São Paulo, São Paulo, Brazil

**Keywords:** Sporotrichosis, Fungal infection, Sporothrix spp, Cutaneous lesion, Osteomyelitis, Children

## Abstract

Sporotrichosis is an infection caused by fungi of the *Sporothrix* complex that can affect both humans and animals. In most cases, it is a benign infection limited to the skin and subcutaneous tissue, rarely disseminating to other organs with systemic involvement. Diagnosis is based on clinical data, epidemiological data, and laboratory tests, with culture being considered the gold standard. The disease generally has a good prognosis and is usually resolved with appropriate antifungal treatment. This case report describes an atypical presentation of sporotrichosis with suppurative nodules and orbital bone involvement in an adolescent followed at the pediatric infectious diseases outpatient clinic of our institution. The patient was followed longitudinally for 12 months due to a lesion on the left upper back with lymphocutaneous involvement, which progressed to osteomyelitis of the left orbit. Deep lesion culture identified *Sporothrix spp*. The sporotrichosis was considered disseminated, and treatment with oral itraconazole for 12 months was initiated. Complete remission of both cutaneous and bone lesions occurred after five months of treatment. The patient was discharged from outpatient follow-up after one year.

## INTRODUCTION

Sporotrichosis is a condition caused by the fungus *Sporothrix*, which affects the skin and subcutaneous tissues and may disseminate throughout the body. The disease affects both humans and animals, especially cats. Transmission occurs mainly through traumatic inoculation of the fungus into the skin. Sporotrichosis presents a broad spectrum of clinical manifestations and it affects individuals regardless of sex, age, or ethnicity^1^.

This descriptive observational study is based on a clinical case report of disseminated sporotrichosis in adolescence with lymphocutaneous and bone involvement in a 13-year-old female middle school patient.

Data were collected during the patient's follow-up at the pediatric infectious diseases outpatient clinic.

### Ethics

The case report was approved by the Research Ethics Committee of the Universidade Federal de Sao Paulo (process Nº CAAE 84969524.2.0000.5505) after the patient provided informed consent and assent forms.

## CASE REPORT

A 13-year-old female residing in the metropolitan region of Sao Paulo city, Brazil, adopted a stray cat. After one year, the animal developed weight loss and lesions on the nose and body before escaping and not returning. Three months later, in November 2023, the patient developed a lesion on the left upper back, initially presenting as a firm erythematous nodule that progressed to a painless ulcer with a necrotic center. Due to lesion progression and the appearance of adjacent nodules, sporotrichosis was suspected and a biopsy was requested. Histopathological examination revealed diffuse suppurative lymphoplasmacytic dermatitis with epidermal hyperplasia, without identification of fungi or parasites.

**Figure 1:**
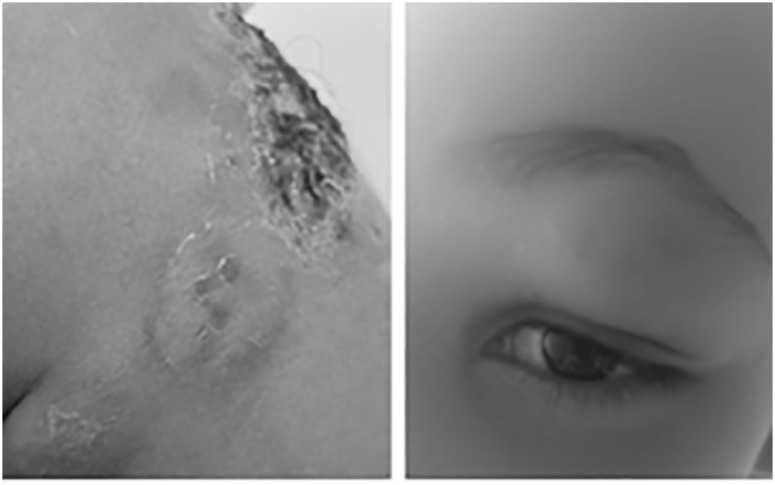
Crusted, painless lesion with a moist ulcerated and necrotic center, progressing to nodular lesions along the lymphatic vessels and a firm, painless nodule on the left upper eyelid measuring 1.5 cm in diameter.

In January 2024, two months after symptom onset, terbinafine 250 mg/day was initiated. After two weeks, a firm nodule appeared on the left upper eyelid, while the lymphocutaneous presentation persisted (Figure 1). In February 2024, the patient was referred to the Pediatric Infectious Diseases Service of the Universidade Federal de Sao Paulo.

On initial evaluation, a painless crusted ulcerated lesion measuring approximately 15 cm was observed on the left upper back, with 3-cm axillary nodular lesions distributed along the lymphatic vessels. Terbinafine was replaced with itraconazole, and the patient was referred to ophthalmology and dermatology departments for deep needle aspiration and tissue sampling.

**Figure 2:**
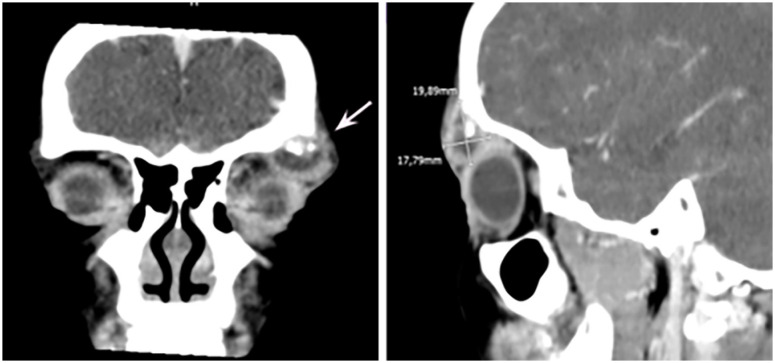
Cranial computed tomography showing an orbital abscess.

Orbital computed tomography demonstrated enlargement and densification of the left periorbital soft tissues, a small subperiosteal collection (5.2 ml), and bone erosion measuring up to 1.4 cm, findings consistent with left periorbital cellulitis associated with osteomyelitis of the orbital roof and post-septal involvement (Figure 2).

Ten days after the initial evaluation, biopsy confirmed the presence of *Sporothrix spp*. The patient was hospitalized with a working diagnosis of disseminated sporotrichosis with lymphocutaneous and osseous involvement and remained hospitalized for 19 days. Given the impossibility of orbital drainage and the limited data available on disseminated sporotrichosis in children, itraconazole was replaced with liposomal amphotericin B, associated with antibiotics due to the possibility of concomitant bacterial infection. The case was reported to the epidemiological surveillance authorities.

On the 14^th^ day of treatment, amphotericin B was replaced with itraconazole due to hypokalemia and hypomagnesemia. During hospitalization, the patient received amphotericin B for 15 days, cefepime for five days, ceftriaxone for seven days, and clindamycin for 14 days. Magnetic resonance imaging performed on the 10^th^ day of hospitalization showed a reduction of the orbital collection to 2 mL. After six weeks of itraconazole therapy, significant improvement of the dorsal and upper eyelid lesions was observed, with central epithelialization and reduction of nodules.

Neurosurgical evaluation revealed no visual field deficits or neurological abnormalities. As imaging demonstrated substantial reduction of the periosteal collection and bone erosion, drainage was not indicated. The patient was discharged for outpatient treatment with itraconazole for one year and amoxicillin-clavulanate for 14 days.

During outpatient follow-up, progressive epithelialization of the dorsal lesion, disappearance of adjacent nodules, and reduction of the eyelid nodule were observed after two months of treatment. After three months of itraconazole therapy, near-complete epithelialization was observed, with only a small central keloid area (0.5 cm) and full resolution of the eyelid nodule. Follow-up magnetic resonance imaging (MRI) at five months confirmed complete resolution of osteomyelitis and absence of orbital abscess. Oral itraconazole was continued until completing 12 months of treatment due to the history of presumed chronic fungal osteomyelitis caused by *Sporothrix*.

Given the rarity of disseminated sporotrichosis with bone involvement during childhood, the patient underwent evaluation by the pediatric immunology team to investigate a possible primary immunodeficiency. Lymphocyte immunophenotyping (including naïve and memory cell subsets) and serum immunoglobulin levels were within normal limits. The dihydrorhodamine test was normal, indicating preserved phagocyte function. Targeted exome sequencing (576 genes associated with immunodeficiency) revealed no known pathogenic variants, although limitations of genetic testing in rare diseases may require future reanalysis.

**Figure 3:**
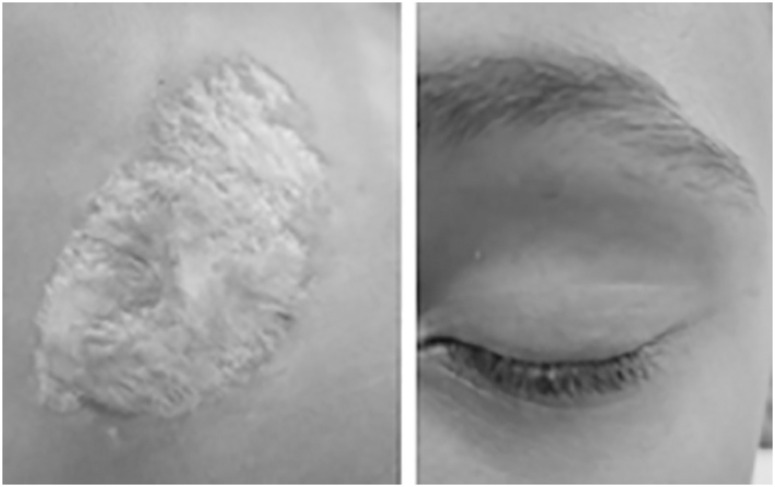
Fully epithelialized dorsal lesion with improvement of the central keloid and complete resolution of the left upper eyelid nodule after one year of treatment.

After 18 months of follow-up and 12 months of specific antifungal therapy, the patient was discharged from the pediatric infectious disease clinic, presenting a hypertrophic scar on the back and no recurrence of cutaneous or orbital lesions (Figure 3).

## DISCUSSION

*Sporothrix spp.* is a dimorphic fungus that exists in a mycelial form at temperatures below 37 °C and in a budding yeast form at temperatures of 37 °C or higher. Different species present distinct geographic distributions: *S. schenckii* predominates in the Americas, Asia, and Africa; *S. brasiliensis* is common in Brazil and is mainly transmitted by cats; whereas *S. mexicana* and *S. globosa* are also described worldwide^2^.

The first isolation of the fungus was performed by Benjamin Schenck in 1898 in the United States. Since then, outbreaks have been documented in several countries, including one in a gold mine in South Africa in 1940 and another among American gardeners in the 1990s^3^. In hyperendemic regions such as Peru, the incidence reaches 98 cases per 100,000 inhabitants, while in Mexico, it is the most prevalent subcutaneous mycosis. In the mountainous regions of Jalisco and Puebla, Mexico, 25 cases per 1,000 inhabitants were reported in 2010^4^.

In Brazil, the first zoonotic case (cat-to-human transmission) was reported in 1955. Beginning in the 1990s, a marked increase in feline cases was observed in Rio de Janeiro, with approximately 2,200 human cases recorded from 1998 to 2009^5^. With the increase in zoonotic transmission associated with contact with cats, atypical presentations, including osteomyelitis, have been increasingly reported^6^.

The urban epidemic from 1997 to 2011 accounted for approximately 5,000 human cases, while more than 5,000 feline cases were reported between 1998 and 2018^7^. Although the disease predominantly affects the Southeast and South regions of Brazil, it has expanded to the Northeast. In 2018, it was reported in more than eight Brazilian states, particularly Rio de Janeiro, Minas Gerais, and Sao Paulo^8^. In Sao Paulo city, 403 human cases had been confirmed by September 2023^9^.

Cat-transmitted sporotrichosis has been widely documented across various Brazilian regions and has recently expanded to neighboring South American countries^10^. Transmission occurs mainly via cutaneous or mucosal inoculation of contaminated plant material or soil and via zoonotic transmission, particularly from scratches or bites from infected cats. Primary pulmonary infection due to inhalation is rare^11^. The incubation period ranges from a few days to three months, and may extend up to six months^9^.

Sporotrichosis is a chronic and polymorphic implantation mycosis that primarily affects the skin and subcutaneous tissue. The most common clinical forms are the fixed cutaneous and lymphocutaneous types, predominantly in areas exposed to trauma such as the face and extremities. Severity depends on inoculum size, depth of inoculation, strain virulence, and host immune status^9,12^.

The fixed cutaneous form is limited to the site of inoculation and is characterized by erythematous nodules and crusts that may progress to ulcers and granulomas, occasionally involving mucosal surfaces. In the lymphocutaneous form, nodules and ulcers develop along lymphatic vessels, producing a "string of beads" appearance. The disseminated form, which is rare, occurs mainly in immunosuppressed individuals, such as people living with HIV, and may involve the bones, lungs, mucosa, eyes, and central nervous system^10^. Conditions such as diabetes, alcoholism, cirrhosis, kidney transplantation, and chronic corticosteroid use also predispose patients to dissemination^6,13,14^.

Ocular manifestations include conjunctivitis, uveitis, choroiditis, and dacryocystitis. Parinaud's oculoglandular syndrome may also occur, with simultaneous ocular and lymph node involvement^9,12^. Extracutaneous forms, resulting from hematogenous dissemination or inhalation, may affect the lungs, bones, joints, liver, and central nervous system, with a risk of sepsis^12^. In a systematic review, Rabello *et al*.^15^ reported 56.1% lymphocutaneous cases, 27.1% fixed cutaneous cases, and 14.3% systemic forms.

Osteoarticular infections are uncommon but have increased due to zoonotic sporotrichosis^13^. Unifocal or localized osteoarticular infection results either from contiguous spread of an overlying cutaneous lesion to the affected area or from deep traumatic inoculation penetrating the skin and reaching the underlying bone or joint. Systemic infection occurs as a result of hematogenous dissemination of *Sporothrix* from various organs or tissues to the bones or joints^6^.

In a systematic review including 211 cases, Ramirez-Soto *et al*.^6^ reported that 53% were localized infections and 47% were systemic infections across 13 countries. Most cases of both localized and systemic infection were reported in the United States and Brazil.

Although rare, intraocular infection may cause inflammation and visual loss, and should be considered in endemic areas. Some patients may also present with exaggerated immune reactions, such as erythema nodosum, erythema multiforme, Sweet's syndrome, and reactive arthritis^13^.

There is no significant sex-related difference in the occurrence of sporotrichosis. The age distribution shows two peaks: school-aged children (30% of cases) and young adults aged 16–35 years (50%). In childhood, the disease mainly affects the face and extremities, presenting with single or multiple lesions and prolonged clinical evolution. Osteoarticular involvement is rare and usually results from direct inoculation or hematogenous dissemination. Pulmonary infections and meningitis are exceptional and occur almost exclusively in immunocompromised individuals^12^.

Diagnosis is confirmed by fungal isolation from clinical samples such as cutaneous lesions, biopsies, secretions, body fluids, or blood^12^. Enzyme-linked immunosorbent assay (ELISA) is a sensitive and specific alternative^16^.

Spontaneous cure is uncommon, and systemic antifungal therapy is usually required^9^. Current guidelines recommend treatment with itraconazole or amphotericin B for osteoarticular sporotrichosis in both immunocompetent and immunocompromised patients. Lower cure rates are generally associated with the severity of osteomyelitis and multifocal involvement, especially in immunocompromised patients, who may not respond adequately to treatment with a single antifungal agent^6^.

Itraconazole is the treatment of choice and is administered orally, with good tolerability and success rates ranging 90% to 100% in cutaneous and lymphocutaneous forms^17^. Clinical response typically occurs within four to six weeks. In severe or disseminated cases, amphotericin B is indicated. Treatment should be continued for three to four weeks after complete lesion healing and may extend up to 60 days. Thermotherapy and cryosurgery are adjunctive options available in specialized centers^9^.

According to the Brazilian Ministry of Health, the overall treatment duration until cure may range from three months to one year. Itraconazole has been shown to be effective and well tolerated, with efficacy rates ranging from 90% to 100% in both cutaneous and extracutaneous forms of sporotrichosis^18^.

Control measures involve epidemiological surveillance, mandatory notification, and health education. Systematic data collection enables estimation of disease prevalence and helps guide public health policies^14^. The close coexistence between humans and cats, combined with feline overpopulation and lack of reproductive control, favors intradomiciliary transmission^19^. Urban factors such as poor sanitation, garbage accumulation, and exposed soil also contribute to disease expansion^8^.

*Sporothrix brasiliensis* plays a central role in the feline sporotrichosis epidemic in Brazil. Early diagnosis and treatment of infected animals are essential to interrupt the chain of transmission. Public health education should emphasize prevention of zoonotic transmission, particularly in hyperendemic areas, warning about the risks of handling cats presenting cutaneous lesions. Individuals at occupational risk—such as gardeners and agricultural or forestry workers—should adopt protective measures when handling soil and vegetation^20^.

Sporotrichosis became a notifiable disease in Brazil under Ordinance Nº 264 of February 17, 2020, which amended Consolidation Ordinance Nº 4/GM/MS of 2017. In Sao Paulo, notification to the national system (SINAN) has been mandatory since 2011. Integrated surveillance between human and animal health sectors is essential for disease control and reduction of *Sporothrix spp*. transmission^8^.

## CONCLUSION

This report contributes to the understanding and clinical management of sporotrichosis in the pediatric population. By thoroughly documenting the clinical presentation, diagnosis, treatment, and outcomes, it highlights the importance of early recognition and appropriate management in improving clinical outcomes and patients’ better quality of life. Additionally, this study provides information that may guide healthcare professionals in managing similar cases and help enhance clinical practice.

## Data Availability

The anonymized dataset generated during this study is available from the corresponding author upon reasonable request.
